# Metabolic diversity in sweet potato (*Ipomoea batatas*, Lam.) leaves and storage roots

**DOI:** 10.1038/s41438-018-0075-5

**Published:** 2019-01-01

**Authors:** Margit Drapal, Genoveva Rossel, Bettina Heider, Paul D. Fraser

**Affiliations:** 1 Biological Sciences, Royal Holloway University of London, Egham, TW20 0EX UK; 2 International Potato Center (CIP), CGIAR Research Program on Roots, Tubers and Bananas, Lima, 12 Peru

**Keywords:** Metabolomics, Genomics

## Abstract

Sweet potato (*Ipomoea batatas*, Lam.) is an important root vegetable in developing countries. After its domestication in Neotropical America, human migration led to the distribution of the sweet potato plant throughout the world. Both leaf and storage root are high in compounds of nutritional value. Yet, the storage roots are of particular value due to their significant content of provitamin A (β-carotene). The breeding effort for elite sweet potato lines led to the reduction of genetic diversity and the potential to improve other traits. The focus of the present study was to assess the metabolic diversity of 27 sweet potato cultivars including landraces and improved varieties. A metabolite profiling approach was optimised for sweet potato leaf and storage root tissue and 130 metabolites identified with three different analysis platforms. The data highlighted a lack of correlation between storage root phenotype and leaf metabolism. Furthermore, the metabolic diversity of storage roots was based on the secondary metabolism, including phenylpropanoids and carotenoids. Three cultivars of three different flesh colouration (yellow, orange and purple) showed a significant difference of the primary metabolism. This data demonstrates the value of metabolite profiling to breeding programs as a means of identifying differences in phenotypes/chemotypes and characterising parental material for future pre-breeding resources.

## Food crops: Assessing the metabolic diversity of sweet potato

Understanding the metabolic variations of different types of sweet potato could help improve their nutritional value. Sweet potato is one of the world’s major food crops but recurrent breeding of high yielding lines is reducing their genetic diversity. A study led by Paul D. Fraser, Royal Holloway University of London, UK, examined the chemical differences between 25 types of sweet potato, including orange and purple varieties. They show that there is little correlation between the chemicals found in the leaves and storage root material and that there are significant differences in metabolism between varieties that produce different pigments in their storage roots. Their findings highlight the importance of metabolite profiling for identifying those varieties that produce the highest levels of starch and health-promoting compounds such as carotenoids.

## Introduction

Sweet potato (*Ipomoea batatas*) is a herbaceous perennial vine, which produces storage roots and edible leaves and can grow on marginal lands. The sweet potato plants have been widely dispersed by humans throughout the world since its domestication in the New World^1,2^. Currently, sweet potato is the sixth most important food crop after rice, wheat, potatoes, maize and cassava. In 2015, 105 million tonnes of sweet potatoes were produced worldwide and 95% thereof in developing countries with China as the lead producer^3^.

Both the leaves and storage roots of sweet potato have a high nutritional value for the human diet. Next to starch which comprises 60% of the dry matter (DM), leaves and storage roots are high in protein, dietary fibre, micronutrients (e.g. iron), vitamins (e.g. vitamin C) as well as bioactive compounds such as carotenoids and phenylpropanoids^4–7^. The main use of sweet potato leaves is as animal feed, as it can be harvested several times throughout the year, whereas storage roots are mainly for human consumption^8^. The storage roots of sweet potato vary in flesh colour from white to orange and to purple, depending on the nature of the pigments produced. The two major pigment classes in sweet potato are carotenoids, cream to orange colouration, and anthocyanins, reddish to bluish purple, and both classes are known for their antioxidant properties. Previous studies highlighted the especially high levels of β-carotene in orange varieties and led to the incorporation of sweet potato into the program to prevent vitamin A deficiency in Africa^9^. Since then, one of the main focus of sweet potato breeding was an increase of starch and DM whilst maintaining high provitamin A levels.

The breeding with recurrent elite lines led to a narrowed genetic pool in the secondary domestication centres, Oceania, Asia and Africa^10^. In addition to this, the main mode of sweet potato dispersal is asexual propagation which leads to a natural reduction of the genetic pool due to a lack of gene exchange^11^. Hence, modern breeding approaches are assessing the diversity of breeding lines and germplasm collection as well as trying to identify the genes involved in starch and carotenoid production through mapping populations^11–14^. Part of the elucidation process for starch and carotenoid biosynthesis is metabolomics, a wholesome approach to the metabolome of the improved tissue. Changes in one metabolite are in general the result of a modulation of many metabolic pathways and might lead to the depletion of other important metabolite(s)^15^. Hence, a metabolite profiling approach has been adapted in the present study to assess the metabolic diversity of leaf and storage root tissue of a sweet potato diversity panel (Table 1). The methods applied include three different platforms to monitor a broad range of metabolites with different biochemical properties.

**Table 1:** List of cultivars included in the diversity panel

Cultivar code	CIP no.	Cultivar name	Country of origin	Biol. status	Root flesh colour
G-0372	401205	Unknown	Mexico	LandRace	Yellow
G-0528	441724^a^	Cuitzeo	Mexico	LandRace	Intermediate orange
G-0615	401527	Unknown	Ecuador	LandRace	Dark purple
G-0882	400473	Unknown	Columbia	LandRace	White
G-1000	401434	Chaco Morado	Venezuela	LandRace	Pale yellow
G-1196	400300	Roxinha	Brasil	LandRace	Pale yellow
G-1320	400177	Aguela Manuchia	Bolivia	LandRace	Pale yellow with purple-red spots
G-1656	400103	Senorita	Argentine	LandRace	Pale yellow with orange spots
G-4615	441141	So 83	Solomon Islands	LandRace	Dark orange
G-5507	401225	Camote	Nicaragua	LandRace	Cream
PT-0005	440001	Resisto	USA	ImpVariety	Intermediate orange
PT-0023	440025	Xushu 18	China	ImpVariety	Cream
PT-0026	440031	Jewel	USA	ImpVariety	Intermediate orange
PT-0045	420014	Jonathan	Peru	ImpVariety	Intermediate orange
PT-0094	420027	Zapallo	Peru	LandRace	Dark yellow with orange ring and spots
PT-0198	440291^a^	Kinabakap	Philippines	LandRace	Cream
PT-0204	440170	Kemb-37	Kenya	LandRace	Dark cream
PT-0309	440175	AVRDC-CN 1038-16	Taiwan	BredLine	Pale yellow with purple spots
PT-0408	440132	Beauregard	USA	ImpVariety	Intermediate orange
PT-0435	440280	85002-103	Tonga	LandRace	Cream
PT-0450	440286	VSP 1	Philippines	ImpVariety	Intermediate orange with purple spots
PT-0451	440287	VSP 3	Philippines	ImpVariety	Intermediate orange
PT-0456	440290	Waimanalao	Philippines	LandRace	Dark cream
PT-0458	440252	Beerwah Gold	Australia	ImpVariety	Intermediate orange
PT-0465	440262	Chin Mi (Eun Mi)	Korea	ImpVariety	Pale orange
PT-0489	440166	Tanzania	Uganda	LandRace	Pale yellow
PT-0561	441755	IB 90.10.20	India	BredLine	Cream

BredLine and ImpVariety represent breeding line and improved variety, the in-progress and final stage of a cultivar, respectively

^a^These cultivars were not included in the metabolite study due to the plants producing fibrous roots

## Results

### Metabolite composition of sweet potatoes

**Fig. 1:**
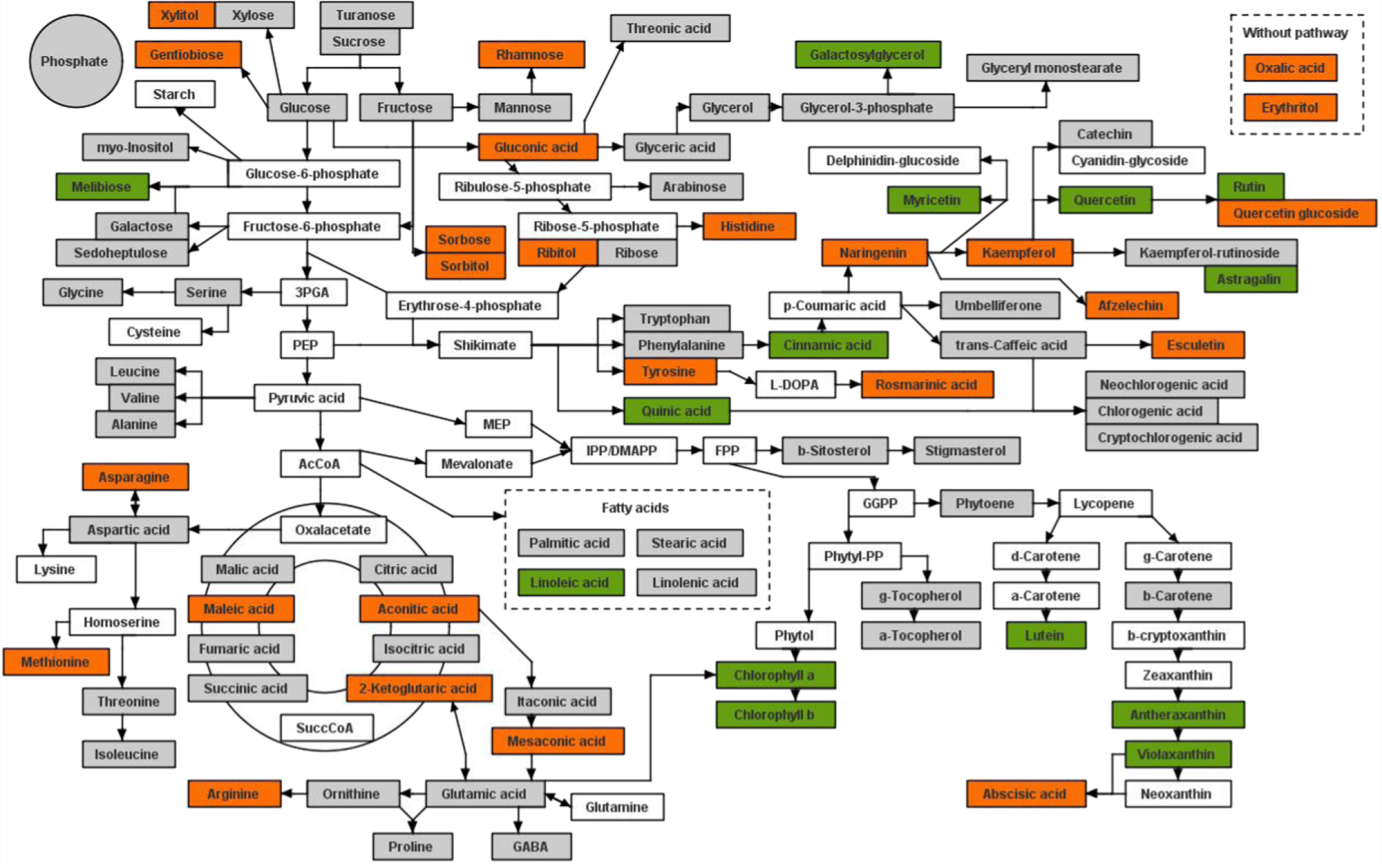
Pathway display of metabolites detected in sweet potato leaf and storage root tissue. The colouration of the metabolites indicates where the metabolite was detected in leaf material (green), storage roots (orange) or both tissues (grey)

The simple extraction method resulted in the detection of 130 identified metabolites in leaf and storage roots (Supplementary File S1). The identified metabolites comprised primary and secondary metabolites of which 66 were detected in both tissue types (Fig. 1, Supplementary File S2). The remaining metabolites were unique to storage root and leaf tissue independently, 47 and 17 components, respectively. A third of all identified metabolites were classified as part of the phenylpropanoid metabolism and included several reoccurring features detected by LC-MS with UV/vis analysis. These features could only be putatively characterised as flavonoids by their spectral properties at 260 nm and were included in the metabolite analysis under the “260 nm” identifier.

The other two-thirds of detected metabolites were classified into the following groups: amino acids, sugars, intermediates of the TCA cycle, sterols, membrane precursors and isoprenoids (IPP derived). The highest proportion of detected metabolite classes on a content basis were represented by sugar with ~85%, followed by phenylpropanoids, intermediates of the TCA cycle and amino acids with 2–5%^7^. Isoprenoids represented 4% in leaf and less than 1% in storage roots and all other compound classes comprised less than 1% in both tissues.

The two cultivars, G-0528 and PT-0198, of the proposed diversity panel did produce fibrous roots and were suspected to carry a viral infection. The analysed material of both leaf and storage roots of these two cultivars were metabolically very different from the other samples, and hence were excluded from any further analysis/discussion of the diversity panel.

### Carotenoid and anthocyanin profiles in leaf and storage root

The main pigments in the storage roots of sweet potato were identified as anthocyanins and carotenoids. The two dominant anthocyanins were identified with LC-MS/MS and comparison to literature as peonidin-3(6″-caffeoyl-6″p-hydroxybenzoyl-sophoroside)-5-glucoside and cyanidin-3(6″caffeoyl-6″-p-hydroxybenzoyl-sophoroside)-5-glucoside^16^. The cultivar G-0615 had the highest levels of both anthocyanins ~3.3 and ~6 mg/g dry weight (DW), respectively. The cyanidin derivative was only detected in one other cultivar, G-1320 with ~1 mg/g DW, whereas the peonidin derivative, known to be the most abundant anthocyanin in sweet potato, was detected in several cultivars, G-1320, G-1196, PT-0309, PT-0450 and PT-0561; all containing ~1 mg/g DW^17^.

**Fig. 2:**
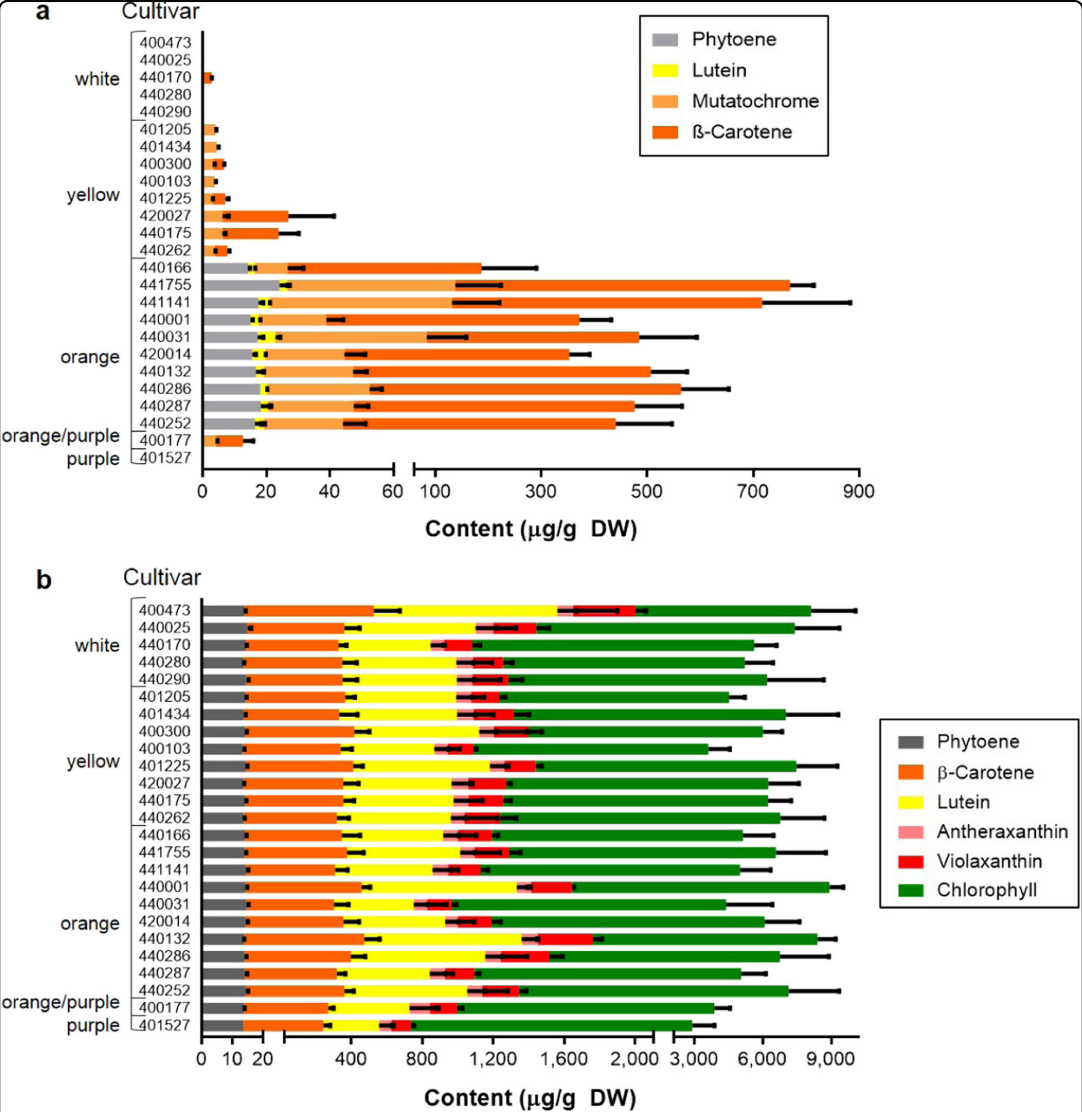
Pigmentation profiles of leaf and storage roots. Carotenoid content of storage root (**a**) and leaf (**b**) tissue analysed as dry weight (DW). Cultivars were grouped into phenotypes by storage root pigmentation, as displayed next to the cultivar codes. Error bars represent the standard deviation of four biological replicates

The carotenoids in storage roots were identified as phytoene, lutein, mutatochrome and β-carotene. Phytoene and lutein were only detected in cultivars with β-carotene levels >160 µg/g DW (Fig. 2a). These ten cultivars also contained the highest levels of mutatochrome, a β-carotene 5,8-epoxide^18^, ranging from 10 to 110 µg/g DW. The cultivar G-4615 contained the highest β-carotene and total carotenoid levels were ~630 and ~800 µg/g DW, respectively. Five cultivars of the diversity panel contained no carotenoids and ten cultivars contained less than 30 µg/g DW of total carotenoids.

In the leaf material, the total content of carotenoids/chlorophylls was 3–9 mg/g DW and chlorophyll constituted the main proportion (Fig. 2b). Neither the β-carotene nor the total carotenoid/chlorophyll content of the leaves could be correlated to the carotenoid content of the storage roots.

For the purpose of this study, the cultivars were grouped according to their carotenoid and specifically the β-carotene content detected in the storage roots: white, yellow, orange, orange/purple and purple including five, nine, nine, one and one cultivars, respectively (Fig. 2a, b).

### Metabolic similarities of sweet potato leaves

**Fig. 3:**
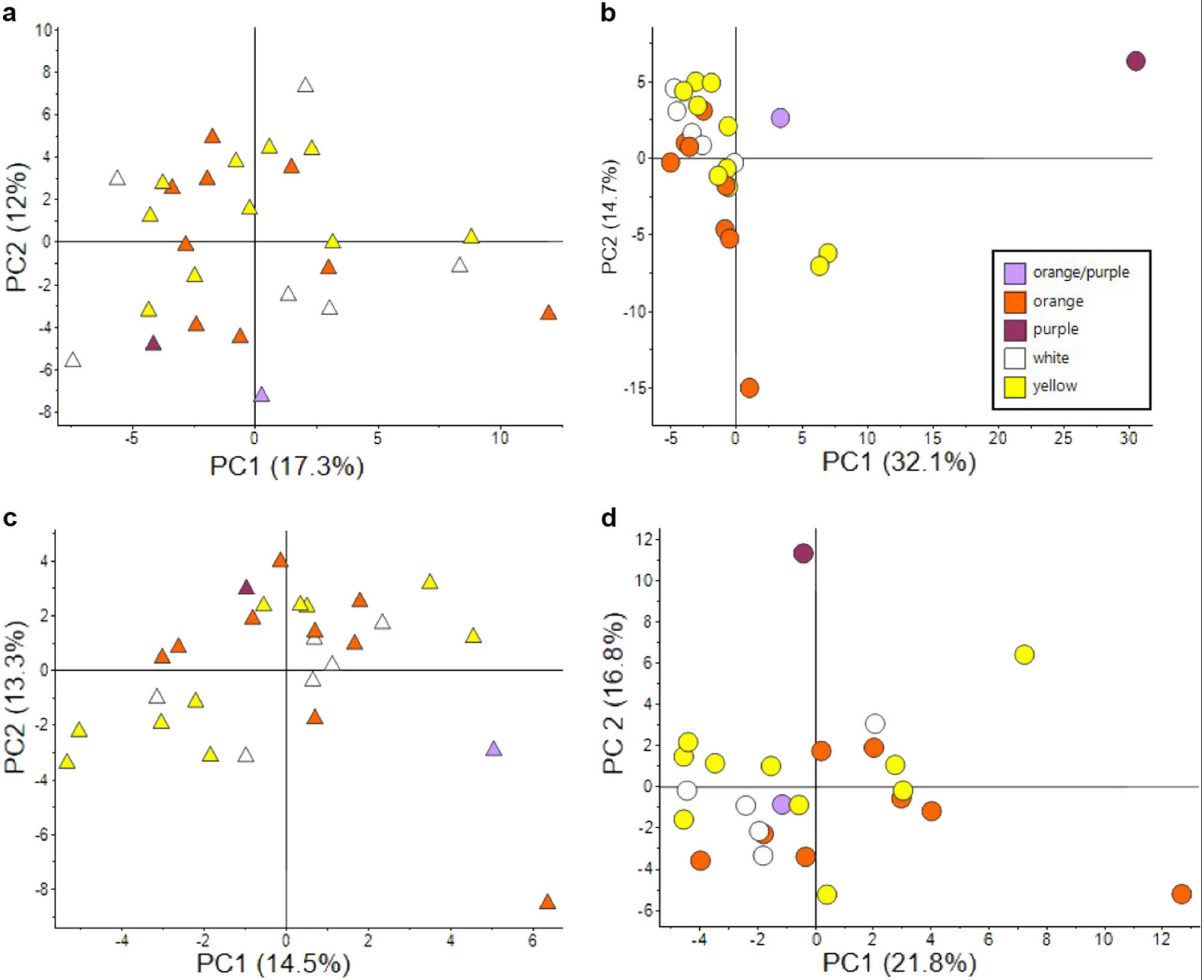
Principal component analysis of metabolite composition of leaf (triangles) and storage root (circles) tissue. The score plots show the variation in the overall metabolite composition (**a**, **b**) and the primary metabolites only (**c**, **d**). Colouration of the cultivars represents the pigmentation of the storage root as displayed in the legend

Principal component analysis (PCA) of the leaf material displayed no separation according to the phenotype of the storage roots. The score plots including all metabolites (Fig. 3a) or only primary metabolites (Fig. 3c) showed mixed clustering of the phenotypes. Analysis of variance (ANOVA) of leaf material detected six metabolites (5% of total metabolite number) significant between phenotypes and about a third of metabolites significant between genotypes (Supplementary File S3). The significant metabolites between phenotypes included mainly phenylpropanoids and a fatty acid, whereas metabolites between genotypes comprised all compound classes including all isoprenoids except phytoene and β-carotene.

### Metabolic diversity of sweet potato storage roots

The score plots of storage roots including all metabolites detected displayed clear cluster trends by phenotype (Fig. 3b), with white phenotypes located between the orange and yellow phenotypes. Contrary to this, the score plot based solely on primary metabolites showed a mixed cluster of all phenotypes (Fig. 3d). In both PCAs, the cultivars G-0615, G-4615 and PT-0309 were located away from the majority of cultivars. G-0615, the purple phenotype, had the highest levels of phenylpropanoids, several sugars and the amino acids aspartic acid, cysteine and GABA. The cultivar G-4615 had the highest carotenoid content and additionally the highest levels of half the amino acids detected. The yellow cultivar PT-0309 had the highest levels of cysteine, histidine and leucine as well as quinic acid and two sugars (Supplementary File S2).

A more detailed analysis of the storage root data by ANOVA indicated significant differences of isoprenoids, phenylpropanoids and sugars between phenotypes, of which isoprenoids were associated with orange cultivars and sugars with the purple phenotype. Half of the metabolites detected were significant between the phenotypes and over 80% of the total metabolites showed significance between the genotypes. The carotenoids had the highest significance values in both cases (Supplementary File S3).

Correlation analysis of the storage root metabolite data highlighted a positive correlation within compound classes, e.g. amino acids, phenylpropanoids and carotenoids (Supplementary Figure S1). Further positive correlations were detected between α-tocopherol and phenylpropanoids; carotenoids and sedoheptulose, citric acid and malic acid; and catechin/epicatechin and anthocyanins. None of the negative correlations were significant.

**Fig. 4:**
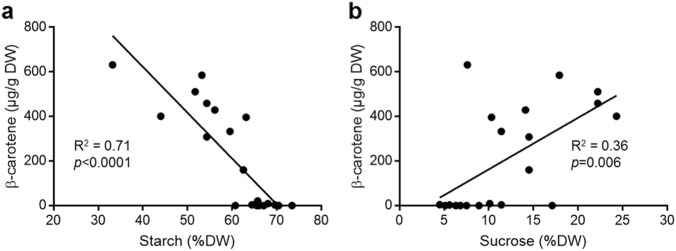
Correaltion between ß-carotene, starch and sucrose content in storage roots. Graph shows the linear regression between ß-carotene measurements and starch (**a**) and sucrose (**b**) content. Linear regression coefficient (R^2^) and significance of the regression are displayed for each graph

The starch, sucrose and β-carotene content of the majority of varieties was evaluated with near-infrared reflectance spectroscopy (NIRS) in a previous crop and compared to the β-carotene measurement with ultra-performance liquid chromatography (UPLC) (Table 2). The β-carotene content established with NIRS showed similar results to the measurements with UPLC, except for some varieties with cream to light orange colour. This was to be expected as the colouration of the powder is comprised of carotenoids and phenolics, which can be clearly distinguished with the more accurate UPLC method. The linear regression between β-carotene and starch content in the storage roots indicated a significant negative correlation, whereas the correlation coefficient for sucrose and β-carotene indicated a low positive correlation (Fig. 4).

**Table 2:** Evaluation of starch, sucrose and carotenoid content in a selected number of cultivars

Cultivar code	CIP no.	Root colour	Starch^a^	Sucrose^a^	β-Carotene^a^	β-Carotene^b^	Mutatochrome^b^
G-1656	400103	Pale yellow with orange spots	62.5	14.5	224.9	160.4	10.3
G-1320	400177	Pale yellow with purple-red spots	68.0	10.1	0.0	8.7	4.0
G-0882	400473	Cream	67.1	7.5	0.0	0.0	0.0
G-0372	401205	Pale yellow	70.4	6.3	88.7	0.0	4.1
G-0615	401527	Dark purple	66.0	8.9	0.0	0.0	0.0
PT-0045	420014	Intermediate orange	44.0	24.3	224.3	401.2	60.5
PT-0094	420027	Dark yellow with orange ring and spots	54.3	14.5	42.4	308.2	25.0
PT-0005	440001	Dark orange	53.2	17.9	576.7	585.2	110.4
PT-0023	440025	Cream	73.4	5.1	0.0	0.0	0.0
PT-0026	440031	Intermediate orange	59.5	11.4	270.5	332.8	21.2
PT-0408	440132	Intermediate orange	54.3	22.2	234.1	459.3	28.1
PT-0204	440170	Cream	69.6	5.6	0.0	2.8	0.0
PT-0309	440175	Pale yellow with purple spots	66.0	4.5	0.0	4.1	3.1
PT-0458	440252	Intermediate orange	63.1	10.3	392.6	396.3	24.8
PT-0465	440262	Pale orange with yellow spots	65.7	N/A	0.0	20.8	6.2
PT-0435	440280	Cream	60.7	17.1	295.2	0.0	0.0
PT-0450	440286	Intermediate orange	51.7	22.2	417.5	511.2	32.2
PT-0451	440287	Intermediate orange	56.1	14.1	324.1	428.8	26.9
PT-0456	440290	Dark cream	65.5	6.8	0.0	0.0	0.0
G-4615	441141	Dark orange	33.2	7.6	506.8	631.9	110.9
PT-0561	441755	Dark yellow	64.4	11.4	58.0	3.9	3.9

Starch and sucrose are displayed as percentage of dry matter and carotenoid content was calculated as μg/g DW

^a^Measurements were taken at CIP

^b^Measurements were taken at RHUL

## Discussion

The present trend in crop breeding is to improve nutritional quality and resilience whilst maintaining high yields^19^. To exploit the natural metabolic variation of sweet potato, the metabolite profiles of a diversity panel representing sweet potato phenotypes were elucidated.

One of the major results of the present study was the lack of correlation between leaf and storage root material, contrary to other crops, e.g. potato and yam^20,21^. This would suggest an independent genetic regulation between two tissue types of the same plant. However, the use of primary metabolites as a differentiator in either tissue displayed no separation between the storage root phenotype, country/region of origin or biological status as reported in genetic studies based on chloroplast and nuclear markers^2,11,22,23^. The metabolite data could reflect the reduced metabolic plasticity of sweet potato storage roots due to multiple recombination events not only in the region of domestication, Neotropical America, but also during the multiple distribution events of sweet potato into Oceania, Asia and Africa^24^. Additionally, the metabolite results support the hypothesis of incorporated transfer DNA from *Agrobacterium* spp. as part of the domestication process, as the landraces with known DNA fragments did not separate from the other cultivars^25^.

Another main discovery was the lack of correlation between carotenoid content and the majority of metabolites in storage roots. The presence of phytoene in orange phenotypes, solely indicates a higher phytoene synthase activity, whereas the correlation of carotenoids with sedoheptulose, citric and malic acid suggests only a minor increase of intermediates/precursors is necessary to support flux into the carotenoid biosynthesis. The metabolite data could therefore be the result of increased *Orange* gene (*Or*) levels, providing more sink space/sites for stable carotenoid storage or the post-translational stabilisation of phytoene synthase. The latter effects could promote increased flux into the carotenoid pathway^26,27^.

Overall, the polyploidy of sweet potato and control of β-carotene levels via at least eight loci poses a challenge to determinate the exact processes involved in higher carotenoids content^13^. Yet, the inverse correlation trend between carotenoid and starch/sucrose content, ascertained on a small subset of the diversity panel (Table 2), has been published previously and supports the hypothesis that β-carotene and starch compete or infer with the formation of macromolecular structures in the same organelles^13,28^. The breeding of the desired starchy, dry and nutritious sweet potato highlighted the compromise between β-carotene and starch/DM content^29,30^. Surprisingly, the sucrose content had a low correlation with β-carotene which would indicate that the carotenoid content and sweetness of the root are unrelated. This is of great benefit for regions preferring sweet potato varieties with low sweetness consumer traits^29^. An example of a sweet potato combining the three traits, low sugar, high starch and high carotenoid levels, is the cultivar PT-0408, an improved variety originating from the USA. This cultivar has a 20% increase in starch content compared to a dark orange cultivar G-4615, and a similar sucrose content to a white cultivar G-0882, but still accounts for ~5% of the RDA for provitamin A per gram DW^31^.

The purple storage roots are in addition to the orange phenotype another source of antioxidants. Purple cultivars such as G-0615 have been reported with 13 times more of total phenolics compared to yellow or orange cultivars, which results in significantly higher radical scavenging activity^6,32^. Rodriguez-Bonilla et al. reported a difference between the leaf tissue of a purple cultivar from other phenotypes, which corresponded to the storage root but not the leaf material of the present study^22^. In both studies, only one purple cultivar was included and prompts a more detailed study of the whole metabolism of several purple phenotypes.

Summarising, the present study demonstrated the successful application of metabolite profiling to assess the metabolic diversity of sweet potato cultivars. The storage root data highlighted three cultivars, G-4615, PT-0309 and G-0615, which differed in their primary as well as secondary metabolite composition and represent suitable parental material for breeding efforts. The lack of metabolic diversity in all other cultivars analysed emphasise the importance of detailed genotyping, phenotyping and trait assessment to prevent further reduction of genetic diversity in elite breeding lines.

## Material and methods

### Plant material

A total of 27 distinct clones were selected from the composite genotyping set to represent the range of diversity inherent in the sweet potato collection preserved in perpetuity at gene bank of the International Potato Center in Lima, Peru. Selection criteria were based on geographic origin, genetic distance and morphological traits such as flesh colour. In vitro plantlets were raised until sufficiently developed to take cuttings. Subsequently, cuttings were planted in 5 L pots in four repetitions and maintained under greenhouse conditions for 9 months in Lima, Peru.

### Measurements of starch, sucrose and β-carotene

Data on starch, sucrose and β-carotene were derived from separate field experiments in 2013 and were provided by the Genoveva Rossel, sweet potato curator at the International Potato Center. NIRS was used to determine starch, sucrose and β-carotene in milled freeze-dried samples of sweet potato storage roots^29^. NIRS calibrations and validation were carried out^33^.

### Sample preparation and extraction

Sweet potato leaf and storage root material was harvested, cleaned and immediately frozen in liquid nitrogen. Storage roots were rinsed in distilled water and cut into cubes before freezing. The frozen material was freeze-dried and ground to fine powder.

For metabolite analysis, a quality control, representing a pool of all samples, was created. All samples including the quality control were weighed (10 ± 0.5 mg) and extracted with a methanol–chloroform protocol^34^. Aliquots of the polar (100 and 650 µl) and the non-polar phase (650 and 700 µl) were dried down after extraction and stored at −20 °C until analysis.

### Metabolite analysis and identification

Aliquots of the non-polar phase were resuspended (leaf 100 µl; storage root 50 µl) in ethyl acetate and acetonitrile (1:9, v/v) and analysed by UPLC. The diluted samples were analysed as previously described, identified by retention time and UV/vis light spectrum and quantified with dose–response curves for each isoprenoid^34,35^.

For gas chromatography-mass spectrometry analysis, the aliquots of the polar phase (100 µl) were dried down with the internal standard d_4_-succinic acid (5 µg) and the non-polar phase (700 µl) with d_27_-myristic acid (10 µg) and derivatised immediately before injection. The GC-MS analysis method included a 10:1 split mode. Identification of metabolites was based on retention time, retention indices and mass spectrum and the area quantified to the internal standard^34,36^

The polar extracts (700 µl) for liquid chromatography-mass spectrometry (LC-MS) were resuspended in methanol/water (100 µl; 1:1, v/v) and an internal standard (genistein, 2.5 µg) added. After LC-MS analysis with a gradient of water/formic acid (0.1%, v/v) and methanol/formic acid (0.1%, v/v), data analysis was performed using R package metaMS with a retention time window match set to 0.5 min^20,36–38^.

### Data analysis and statistical modelling

Relative quantified data matrices were subjected to PCA using Simca P 13.0.3.0 (Umetrics, Sweden). Non-parametric one-way ANOVA after auto-scaling was performed using MetaboAnalyst 3.0^39^. UPLC results were displayed as bar charts using GraphPad Prism 7.02 (CA, USA). Metabolites were displayed as biochemical pathways constructed with BioSynLab software (www.biosynlab.com).

## Electronic supplementary material

Suppl Figure 1

Supplementary File S1

Supplementary File S2

Supplementary File S3
