# Supplementary figures and images for: Metabolic diversity in sweet potato (Ipomoea batatas, Lam.) leaves and storage roots
Source: Hortic Res. 2019 Jan 1;6:2. doi: 10.1038/s41438-018-0075-5 (PMC6312539; doi:10.1038/s41438-018-0075-5)

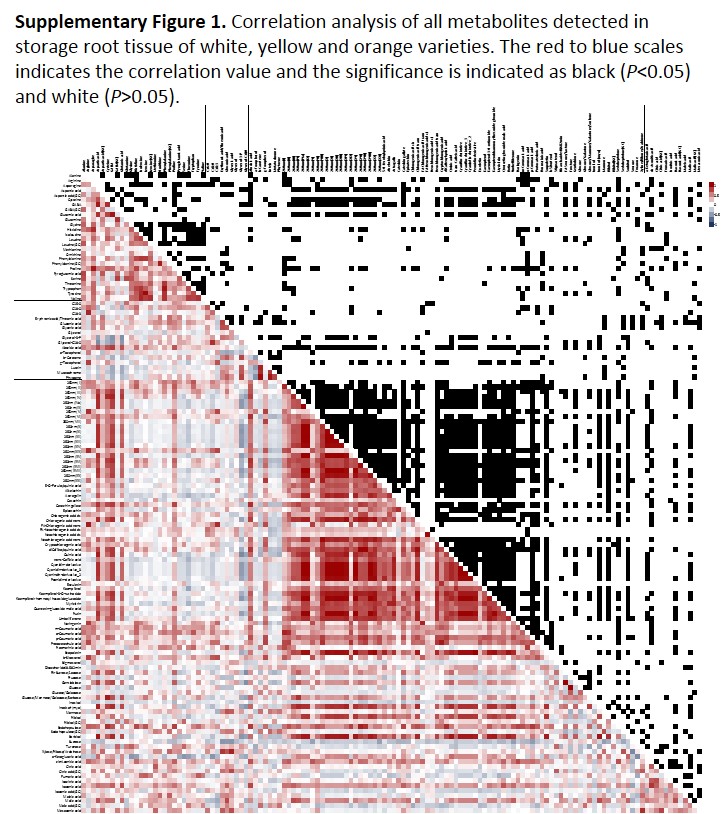

Supplement: Supplementary file 1 — Suppl Figure 1 [file 41438_2018_75_MOESM1_ESM.jpg]
